# Retraction Note: Target receptor identification and subsequent treatment of resected brain tumors with encapsulated and engineered allogeneic stem cells

**DOI:** 10.1038/s41467-025-57909-0

**Published:** 2025-03-21

**Authors:** Deepak Bhere, Sung Hugh Choi, Pim van de Donk, David Hope, Kiki Gortzak, Amina Kunnummal, Jasneet Khalsa, Esther Revai Lechtich, Clemens Reinshagen, Victoria Leon, Nabil Nissar, Wenya Linda Bi, Cheng Feng, Hongbin Li, Yu Shrike Zhang, Steven H. Liang, Neil Vasdev, Walid Ibn Essayed, Pablo Valdes Quevedo, Alexandra Golby, Naima Banouni, Anna Palagina, Reza Abdi, Brian Fury, Stelios Smirnakis, Alarice Lowe, Brock Reeve, Arthur Hiller, E. Antonio Chiocca, Glenn Prestwich, Hiroaki Wakimoto, Gerhard Bauer, Khalid Shah

**Affiliations:** 1 Center for Stem Cell and Translational Immunotherapy (CSTI), Brigham and Women’s Hospital, Harvard Medical School, Boston, MA USA; 2 Department of Neurosurgery, Brigham and Women’s Hospital, Harvard Medical School, Boston, MA USA; 3 Department of Radiology, Massachusetts General Hospital, Harvard Medical School, Boston, MA USA; 4 Department of Medicine, Brigham and Women’s Hospital, Harvard Medical School, Boston, MA USA; 5 Department of Renal Medicine, Brigham and Women’s Hospital, Harvard Medical School, Boston, MA USA; 6 Department of Neurology, Brigham and Women’s Hospital, Harvard Medical School, Boston, MA USA; 7 UC Davis Institute for Regenerative Cures, Davis, CA USA; 8 Department of Pathology, Brigham and Women’s Hospital, Harvard Medical School, Boston, MA USA; 9 Harvard Stem Cell Institute, Harvard University, Cambridge, MA USA; 10 Amasa Therapeutics Inc., 1 Harmony Lane, Andover, MA USA; 11 Department of Medicinal Chemistry, College of Pharmacy University of Utah, Salt Lake City, UT USA; 12 Department of Neurosurgery, Massachusetts General Hospital, Harvard Medical School, Boston, MA USA; 13 Present Address: Department of Pathology, Microbiology, and Immunology, University of South Carolina School of Medicine, Columbia, SC USA; 14 Present Address: Department of Pathology, Stanford University, Stanford, CA USA; 15 Present Address: Washington State University Health Sciences, Spokane, WA USA

Retraction to: *Nature Communications* 10.1038/s41467-022-30558-3, published online 19 May 2022

The authors have retracted this article. After publication, concerns were raised regarding highly similar images between the figures presented in this article and a number of other sources, specifically:

- Fig. 1e GBM8 DR5 image appears highly similar to Fig. 1 Tu+Met Casp-1 in ref. ^1^;
- Fig. 1e GBM18 DR4 image appears highly similar to Fig. 3b HNSSC (PD) in ref. ^1^;
- Fig. 1f GBM31R mCherry image appears highly similar to Fig. 5f AGFP+MSC-GFP mCherry image in ref. ^2^;
- Fig. 1f GBM8 DR5 image appears highly similar to the BCHE Antibody image on the FisherScientific website ref. ^3^;
- Fig. 1f GBM8 DR4 image appears highly similar to Fig. 5c in ref. ^4^;
- Fig. S2a both images appear highly similar to human bone-derived mesenchymal stem cell images on the ScienCell website ref. ^5^;
- Fig. S4b Brain Untreated and EnMSC-Bif50 Week 2 images appear highly similar to Fig. 4c AZD9291 15 and 30 mg/kg in ref. ^6^, respectively;
- Fig. S4b Lung and Kidney EnMSC-Bif500 Week 4 and 8 images appear highly similar to Fig. 8a Lung (HA-VES7/DOX and DOX-Sol) and Kidney (HA-VES4/DOX and HA-VES12/DOX) images in ref. ^7^;
- Fig. S4b Brain EnMSC-Bif500 Week 8 image appears highly similar to Fig. 6a middle image in ref. ^8^;
- Fig. S4b Lung Untreated image appears highly similar to Fig. 2a Naturally aging group Lung image in ref. ^9^;
- Fig. S4b Lung EnMSC-Bif50 Week 2 image appears highly similar to Fig. 4 Lung WT image in ref. ^10^;
- Fig. S5a EnMSC-GFP Day 1, EnMSC-Bif Day 42, and EnMSC-Bif+GCV Day 1 images appear highly similar to Fig. 6e d7 and d15 A172-S TRAIL images in ref. ^11^.

The authors have reviewed the data and confirmed that the images were reused incorrectly and without appropriate references.

Deepak Bhere, Pim van de Donk, Victoria Leon, Naima Banouni, Reza Abdi, Arthur Hiller, Glenn Prestwich, Hiroaki Wakimoto, and Khalid Shah agree to this retraction. The publisher has been unable to obtain the current email addresses for David Hope, Jasneet Khalsa, Esther Revai Lechtich, Hongbin Li, Walid Ibn Essayed, Pablo Valdes Quevedo, and Gerhard Bauer. None of the other authors have responded to any correspondence from the publisher about this retraction.
